# Artificial Intelligence in Prehospital Tele-Emergency Medicine: A Survey of Acceptance and Attitudes

**DOI:** 10.3390/healthcare14172753

**Published:** 2026-08-29

**Authors:** Nadezhda Durdova, Mathias Schmidt, Hanna Schröder, Pia Thoma, Dominik Groß, Saskia Wilhelmy

**Affiliations:** 1 Institute for History, Theory & Ethics of Medicine, University Hospital, RWTH Aachen University, 52074 Aachen, Germany; maschmidt@ukaachen.de (M.S.); dgross@ukaachen.de (D.G.); saskia.wilhelmy@tu-dresden.de (S.W.); 2 Aachen Institute for Rescue Management & Public Safety, University Hospital, RWTH Aachen University & the City of Aachen, 52074 Aachen, Germany

**Keywords:** artificial intelligence, tele-emergency medicine, acceptance, clinical decision support system, ethics

## Abstract

**Background/Objectives:** Artificial intelligence (AI) is increasingly being used in medicine to improve the efficiency, accuracy, and timeliness of patient care. In prehospital tele-emergency medicine, AI also has the potential to address various challenges and support tele-emergency physicians. This study focuses on a specific AI-based decision support system being developed for use by practitioners. While technical feasibility is a prerequisite for the successful implementation of this system, social, ethical, and patient-centered considerations are equally important. **Methods:** The acceptance and perceptions of the German public, as potential patients, regarding the implementation of a specific AI system for prehospital tele-emergency medicine were assessed through an online survey. The results provide qualitative and quantitative insights into participants’ attitudes and acceptance. **Results:** The perceived advantages and disadvantages of implementing AI in prehospital tele-emergency medicine, as seen by potential patients, were identified, along with moderate acceptance of the technology. Participants emphasized the importance of different factors, including time efficiency, safety, AI recommendation accuracy, responsible system use, data and legal protection, technical stability, and positive impact on physicians’ work and patient outcomes. **Conclusions:** The findings enhance understanding of public perceptions regarding the use of AI in prehospital tele-emergency medicine and provide a valuable bioethical foundation for guiding the responsible integration of AI into tele-emergency care.

## 1. Introduction

Artificial intelligence (AI) has become an increasingly integral part of everyday life, with individuals encountering it repeatedly, either consciously or unconsciously [1,2]. It offers numerous opportunities to accelerate or facilitate processes across various domains, including healthcare [3]. In the medical context, intelligent systems are being progressively implemented with the promise of improving the efficiency, accuracy, and timeliness of patient care [4,5,6].

Perceptions and acceptance of artificial intelligence (AI) in medicine vary widely. While some patients do not trust medical AI and fear the unknown, others believe it could improve diagnostic accuracy and save time [7]. These varying perspectives, as well as acceptance of AI in healthcare contexts, are associated with multiple factors, including demographic characteristics such as age [8,9,10], gender [10,11,12], educational level [11], occupation [13], technical affinity and attitudes toward technology [14,15], previous experience with AI [16], and trust in the reliability and safety of specific AI applications [10,17]. Overall, the use of AI in medicine is widely supported, especially when used as an assisting tool rather than a substitute for physicians [18]. While patients see the advantages of AI in its accuracy, efficacy, and ability to process large volumes of information, they also believe it could not replace physicians, as it lacks human-like empathy and interactional skills [19]. Acceptance of medical AI also appears to depend on the specific application and degree of task complexity. Recent studies indicate that the use of AI for diagnosis or to support treatment is generally accepted, but its use in medical triage, for example, is rejected [11], with a higher level of trust placed in medical triage conducted by humans [20].

To date, AI applications are available in nearly all medical fields [21]. For example, AI-based clinical decision support systems can provide healthcare professionals with information and recommendations to support patient care. Recent research emphasizes that the successful implementation of such systems requires considering factors beyond predictive performance, including transparency, explainability, integration into clinical workflows, and appropriate human oversight [22,23].

In Germany, AI is expected to play a key role in addressing challenges in the healthcare system in the future, such as staff shortages and the simultaneously rising number of patients with multiple and complex conditions [24]. Such challenges are being observed in various medical fields in Germany, including prehospital tele-emergency medicine, where the main challenges are related to the growing number of emergency missions for tele-emergency physicians [25]. The potential use of AI in prehospital emergency medicine requires specific framework conditions, as it is characterized, for example, by the need for time-critical decision-making, the challenges of dealing with limited and rapidly changing information, and the high cognitive demands of the tasks involved [26,27]. Errors or delays can have a detrimental impact on the patients’ outcomes [28]. Therefore, reliable and high-performing models are particularly important for AI applications in prehospital settings [26].

This study focuses on an AI-based decision support system being developed for tele-emergency physicians as part of the German KIT^2^ project (“KI-unterstützter Telenotarzt (KIT^2^)”, English: AI-supported tele-emergency physician), funded by the German Federal Ministry of Research, Technology and Space (grant number 13N16401). The potential integration of AI into prehospital tele-emergency medicine aims to assist tele-emergency physicians in a variety of tasks, such as generating preliminary diagnoses, recommending medications and interventions, and identifying the most suitable hospital for a patient based on their needs and the availability of resources. The high stakes of these functions raise specific questions regarding accountability and regulation [29]. Accuracy is also a vital concern when AI recommendations influence medical decisions made under time pressure [30].

While technical feasibility is crucial for successful implementation, social, ethical, patient- and user-centered considerations are equally important [31]. Prior research within the KIT^2^ project addressed the perspectives of tele-emergency physicians, the future primary users of the AI system [32]. This study shifts the focus to the views of potential patients, exploring their acceptance and perspectives regarding the implementation of a specific AI system within the German prehospital tele-emergency medical system, thereby addressing a critical gap in the literature. By focusing on this niche yet crucial area, the study contributes to a better understanding of how patients perceive AI when it is deployed in time-critical tele-emergency scenarios.

## 2. Materials and Methods

### 2.1. Study Design and Procedure

We conducted an online survey using SoSci Survey [33] between August and December 2023. This paper reports the survey components relevant to the present research questions, namely the acceptance of AI-supported prehospital tele-emergency medicine and its perceived advantages and disadvantages. Quantitative items assessed acceptance and prior exposure to common AI applications, whereas open-ended questions explored perceived benefits and concerns.

### 2.2. Acceptance Definition for the Study

To address the objectives of this study, it was first necessary to define the term “acceptance” in relation to AI-supported tele-emergency medicine. The definition of acceptance used in this study was derived from Schmidt et al. [34], who define technical acceptance as “the user’s willingness to apply a concrete technology or technical system in a concrete situation, or—specifically referring to the perspective of patients, who cannot be considered as active users—to allow it to be applied to them”. This definition is consistent with further available definitions of technology acceptance [35,36,37,38]. Therefore, in the context of this study, the term “acceptance” refers to the willingness of the German population to be treated by an AI-supported tele-emergency physician during a rescue operation.

### 2.3. Participants and Recruitment

Adults aged 18 years or older from the general German public, considered potential patients, were eligible to participate. Participants were recruited using a convenience sampling approach. The questionnaire was distributed through various communication channels, including institutional websites, the website of a popular science magazine, the email distribution list of a patient organization, social media, and physical flyers.

### 2.4. Measures

#### 2.4.1. Prior Use and Satisfaction with AI Systems

A brief, simple definition of AI was provided to introduce participants to the study topic and to ensure that they had at least a basic understanding of AI before proceeding with the questionnaire: “Artificial Intelligence (AI) is the term used to describe technologies that imitate the intelligent behavior of humans. Similar to a human, an AI should learn with the help of data and experience to independently find solutions to problems in order to support its users as well as possible.” [39].

**Figure 1:**
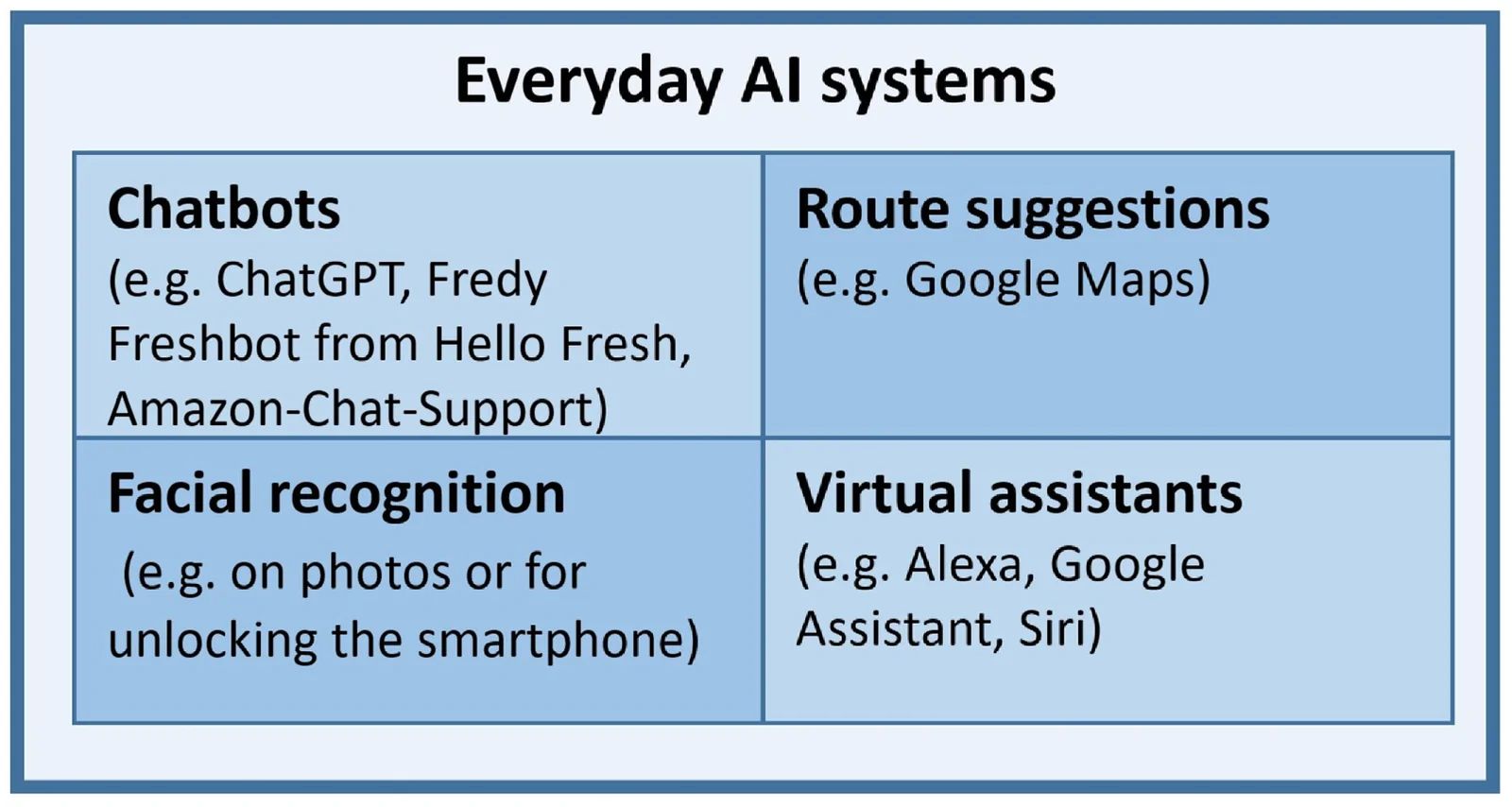
Everyday AI systems included in the questionnaire.

Subsequently, two scales were used to measure participants’ use of AI systems in everyday life and their satisfaction with those systems (Figure 1; Supplementary File S1). AI use was assessed using a four-point frequency scale ranging from “daily” to “yearly”, while satisfaction was assessed using a four-point Likert scale ranging from “satisfied” to “dissatisfied”. Both scales additionally included the response options “never used” and “no response”.

#### 2.4.2. Acceptance of AI-Supported Tele-Emergency Medicine

To ensure a comparable level of knowledge among all participants, a brief description of the tele-emergency physician system was provided: “The tele-emergency physician system allows paramedics to communicate digitally with a remote emergency physician during a rescue operation (tele-emergency physician). The tele-emergency physician receives all relevant information about the patient in real time (e.g., temperature, blood pressure, medication, allergies, etc.). There is also the option to receive a live video stream from the ambulance. This enables the tele-emergency physician to gain an overview of the emergency situation and the patient remotely and to provide support to the paramedics on site” [40].

Participants also received a brief overview of an AI-based decision-support system for tele-emergency physicians being developed within the KIT^2^ project: “Imagine an artificial intelligence (AI) that supports the tele-emergency physician in their work by suggesting possible diagnoses, treatment options, and options for further patient transportation. The suggestions are based on information entered into the AI system by the tele-emergency physician (e.g., temperature, blood pressure, medication, allergies, etc.). The system is not intended to replace the tele-emergency physician in their work but rather to offer them support. All decisions during the rescue operation are still made by and remain the responsibility of the tele-emergency physician”.

**Figure 2:**
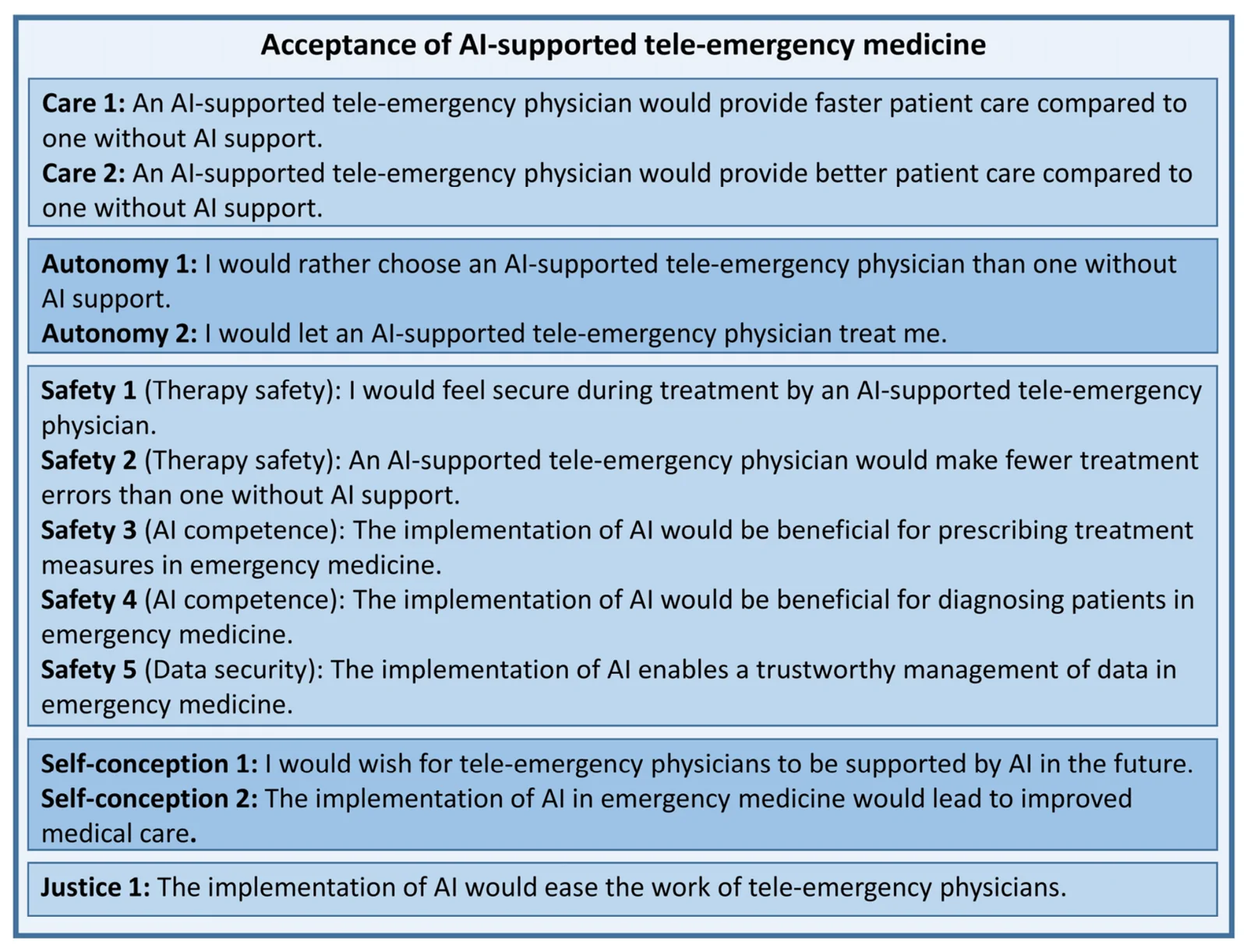
Items for assessing participants’ acceptance of AI-supported tele-emergency medicine. All items are rated on a four-point Likert scale from “strongly agree” to “strongly disagree” with the additional option “no response”.

Participants were then asked to indicate their level of agreement with twelve statements concerning AI support for tele-emergency physicians and the implementation of AI in tele-emergency medicine (Figure 2; Supplementary File S2). The order of these statements was randomized for each participant to avoid sequence effects [41]. The scale used was based on the Model for Ethical Evaluation of Socio-Technical Arrangements (MEESTAR) [42,43].

Five MEESTAR dimensions for ethical evaluation (care, autonomy, safety, self-conception, and justice) were operationalized as positive statements. Care was defined as the ability to care for others, especially in situations in which they are unable or only partially able to care for themselves [44]. Autonomy was conceptualized as the individual’s freedom of decision [44]. The concept of safety was divided into three categories: AI competence, therapy safety, and data security [43]. Self-conception was understood as the perception of potential changes, including those in professional roles due to technological progress [45]. Justice was defined as fairness and the absence of detriment to the tele-emergency physician [46].

The instrument for ethical evaluation, MEESTAR [42,43], formed the basis for the development and structuring of the acceptance scale of the questionnaire. Nevertheless, from a practical standpoint, some of the resulting items correspond to the constructs described in the Technology Acceptance Model (TAM) [47] and the Unified Theory of Acceptance and Use of Technology (UTAUT) [48]. In particular, the items concerning faster and better patient care (“Care 1” and “Care 2”), improved medical care (“Self-conception 2”), diagnostic and therapeutic benefits (“Safety 2”, “Safety 3”, and “Safety 4”), and reduced physician workload (“Justice 1”) are conceptually related to perceived usefulness in TAM and performance expectancy in UTAUT. The items assessing participants’ preference for an AI-supported tele-emergency physician and their willingness to receive treatment from one (“Autonomy 1” and “Autonomy 2”) are closely related to behavioral intention to use the system [48] or, as in the KIT^2^ case, to allow it to be used on one [34].

#### 2.4.3. Perceived Advantages and Disadvantages

To qualitatively assess participants’ perceptions of AI in prehospital tele-emergency medicine, three open-ended questions were posed: (1) “What advantages do you see in using AI in the tele-emergency physician system?”; (2) “What disadvantages do you see in using AI in the tele-emergency physician system?”; and (3) “Is there anything else on this topic that you would like to share with us?”.

#### 2.4.4. Demographic Information

Respondents were invited to provide information about their sociodemographic characteristics, including gender, age, and level of education, as well as whether they have had any previous experiences with emergency care, for example, as a patient or as an accompanying person.

### 2.5. Pretest and Data Cleansing

A pretest with individuals who were not involved in the main study was conducted (N = 22) to ensure comprehensibility, appropriate item wording, and technical functionality. Minor adjustments to item wording and punctuation were subsequently made to create the final items used in this study.

A total of 570 individuals participated in the online survey. Based on predefined exclusion criteria, one participant under 18 years of age, the fastest 10% of respondents (N = 57) as an indicator of insufficient engagement [49,50,51], and 15 boxplot-identified outliers on the AI-supported tele-emergency medicine acceptance scale [52] were excluded. This resulted in a final analytic sample of 497 participants.

After data cleansing, the mean completion time of the study was 7.96 min (SD = 3.03 min, range: 4.03–20.63 min). The statistical analysis was carried out using IBM SPSS Statistics (Version 29) for Windows [53]. Qualitative data were analyzed manually according to Mayring [54].

## 3. Results

### 3.1. Sample Characteristics

The analyzed sample consisted of slightly more women than men, with a mean age of 41 years. Most participants reported having a high school diploma or a university degree as their highest level of education. Around half of the participants reported previous experience with emergency medical care (Table 1).

**Figure 3:**
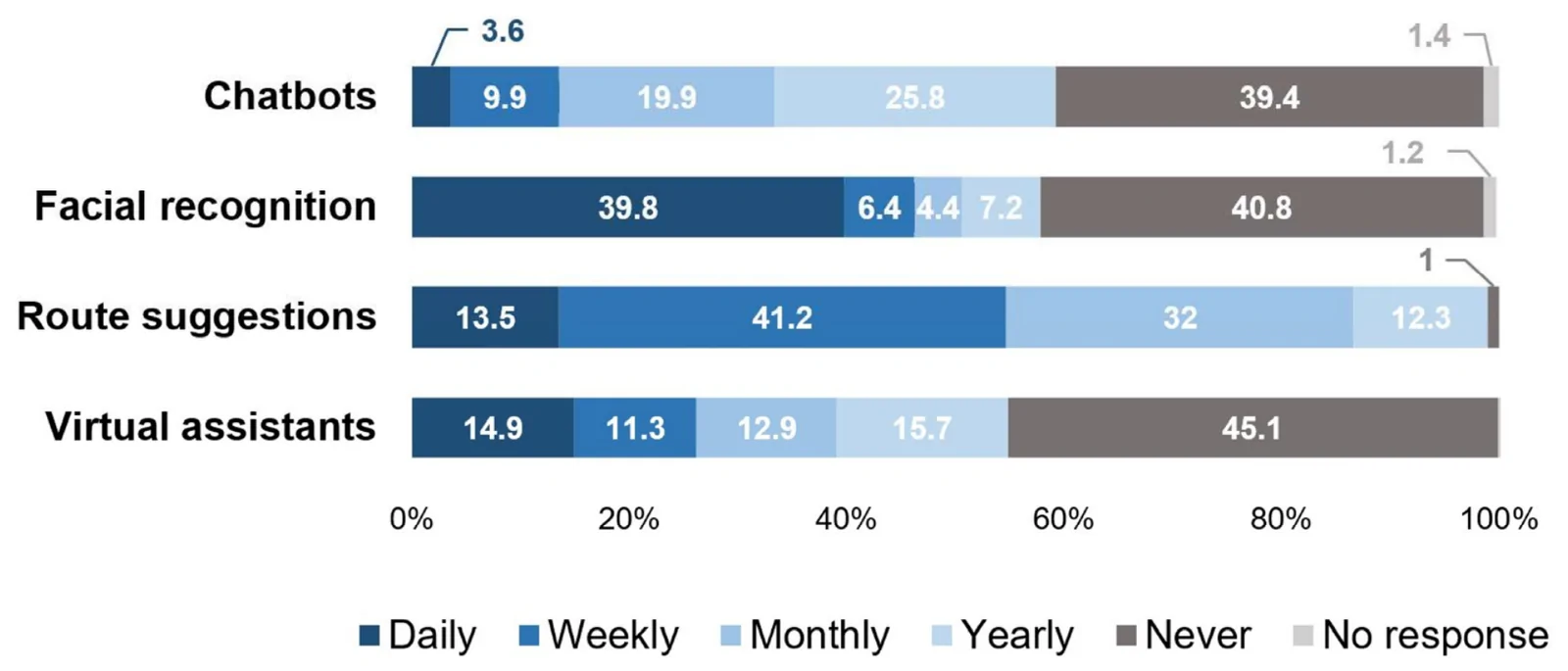
Self-reported average use of AI systems (N = 497).

**Figure 4:**
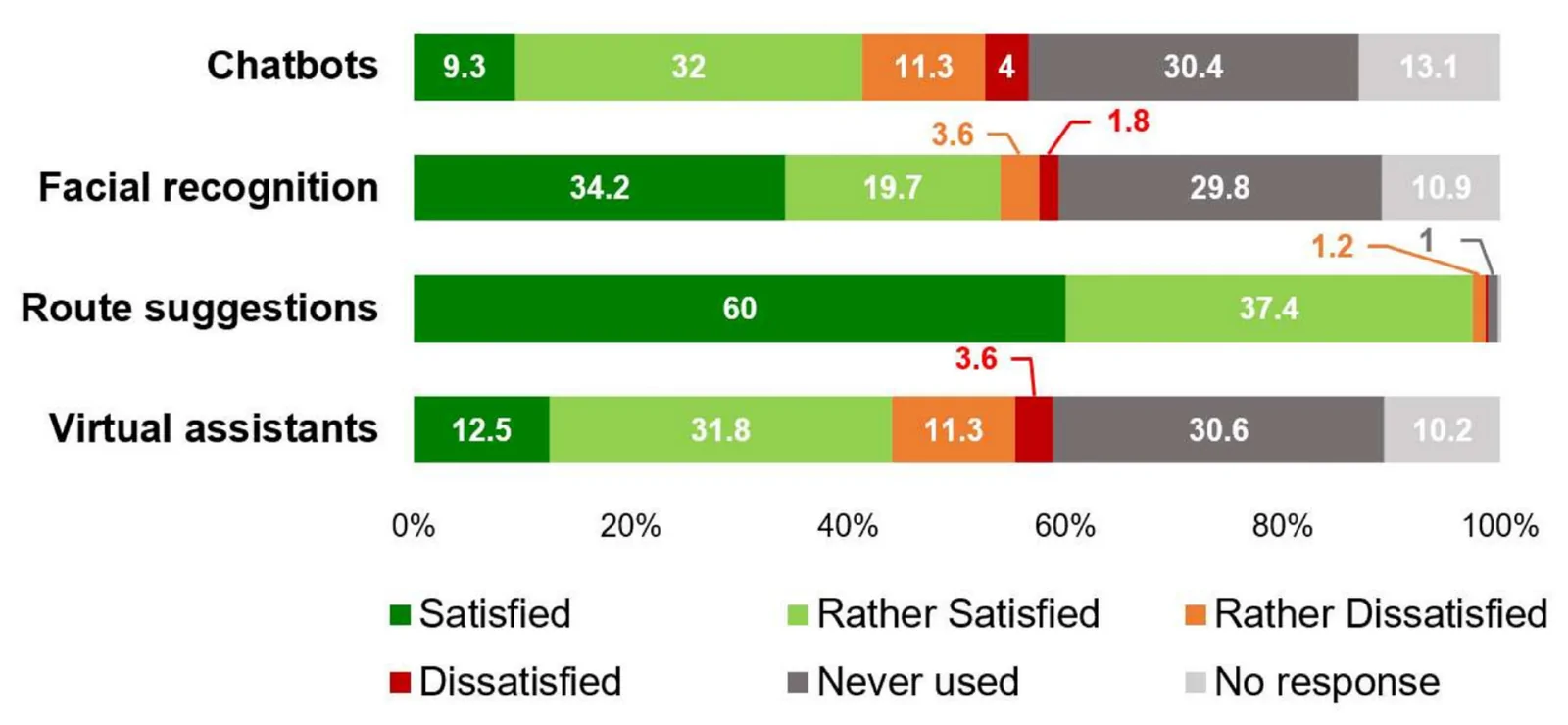
Self-reported satisfaction with the use of AI systems (N = 497).

Regarding prior exposure to everyday AI applications, almost all participants reported using route suggestions at least once per year (99%). The use of virtual assistants at least yearly was reported by 54.8% of participants, while 57.8% reported using facial recognition and 59.2% reported using chatbots (Figure 3). Satisfaction was highest for route suggestions and lowest for chatbots (Figure 4).

### 3.2. Acceptance of AI in Prehospital Tele-Emergency Medicine

**Figure 5:**
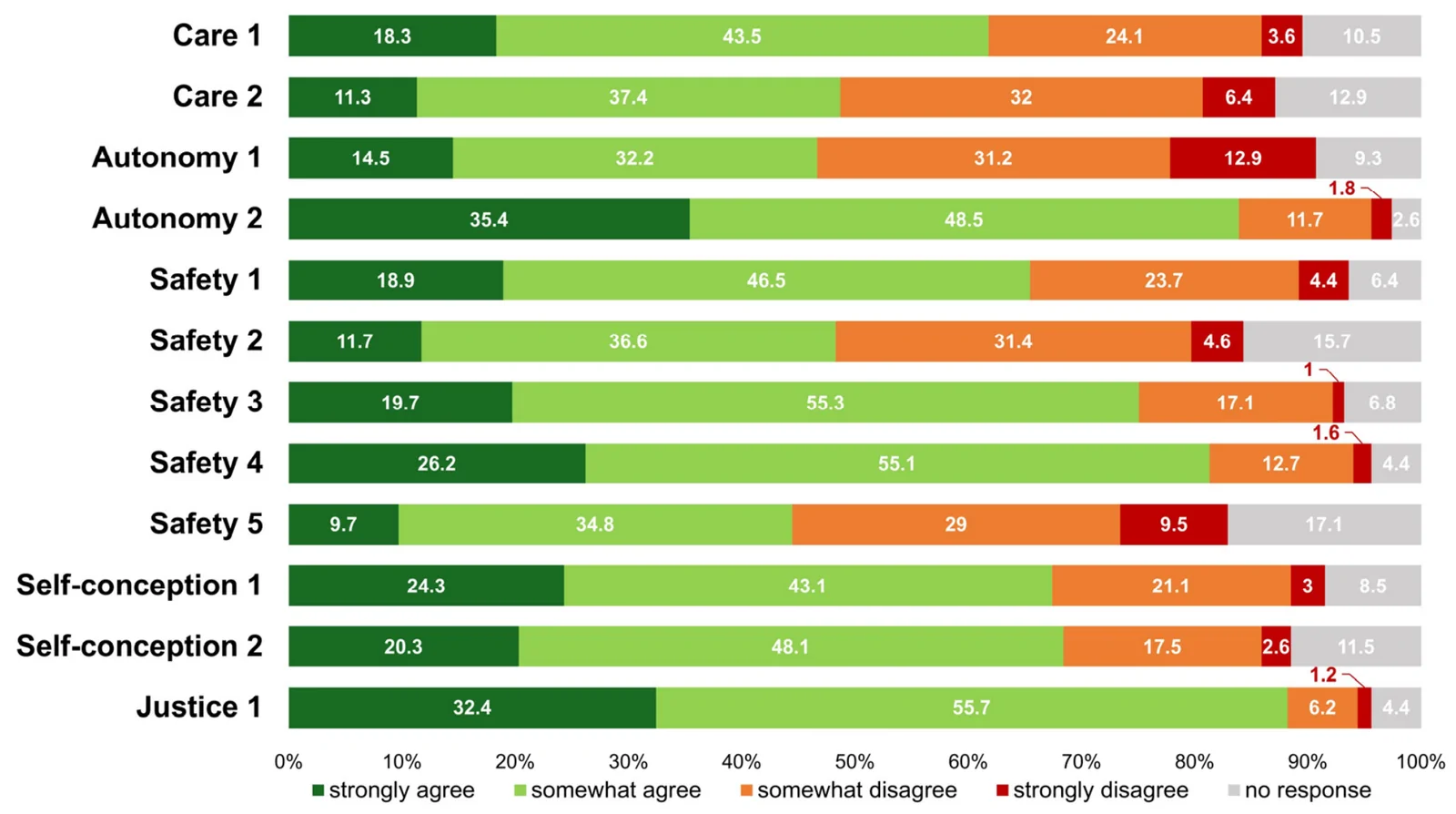
Agreement score with the items measuring acceptance of AI-supported tele-emergency medicine (N = 497).

Item-level response distributions for the 12 items of the acceptance scale are shown in Figure 5. The overall agreement with the 12 items of the acceptance scale on AI-supported tele-emergency medicine was moderate (M = 2.90, SD = 0.60, N = 312, range: 1.33–4.00, 1 = “strongly disagree” to 4 = “strongly agree”). The internal consistency of the scale was excellent (α = 0.93) [55].

Women showed lower acceptance of AI-supported tele-emergency medicine (M = 2.82, SD = 0.60, N = 178) than men (M = 3.02, SD = 0.59, N = 133), t(309) = 2.93, *p* = 0.004. Age showed a small positive association with acceptance, r(310) = 0.133, *p* = 0.019. There was no significant difference in acceptance between people who already had experience with emergency medical care (M = 2.89, SD = 0.59, N = 144) and those who did not (M = 2.87, SD = 0.60, N = 111), t(253) = 0.30, *p* = 0.77. Acceptance did not differ significantly by educational attainment, Welch’s F (2, 83.92) = 2.18, *p* = 0.120.

The use of different AI systems was weakly positively associated with acceptance (Table 2), and satisfaction with route suggestions was also positively associated with acceptance (Table 2).

Two separate multiple linear regression analyses were conducted to examine predictors of acceptance of AI in tele-emergency medicine. The first model, which included demographic characteristics and previous AI use, was statistically significant, F(6, 296) = 9.88, *p* < 0.001, and explained 16.7% of the variance in acceptance (R^2^ = 0.17, adjusted R^2^ = 0.15). Age (B = 0.01, SE = 0.002, β = 0.24, t = 4.04, *p* < 0.001), average use of route suggestions (B = 0.11, SE = 0.04, β = 0.17, t = 2.96, *p* = 0.003), average use of facial recognition (B = 0.04, SE = 0.02, β = 0.12, t = 2.07, *p* = 0.039), and average use of chatbots (B = 0.12, SE = 0.03, β = 0.25, t = 4.10, *p* < 0.001) were significant positive predictors of acceptance. Gender (B = 0.10, SE = 0.07, β = 0.08, t = 1.44, *p* = 0.151) and average use of virtual assistants (B = −0.01, SE = 0.02, β = −0.03, t = −0.44, *p* = 0.662) were not significant.

The second model, which included demographic characteristics and AI satisfaction, was not statistically significant, F(6, 104) = 1.85, *p* = 0.096, R^2^ = 0.10, adjusted R^2^ = 0.04. None of the individual predictors were statistically significant: age (B = 0.01, SE = 0.01, β = 0.18, t = 1.73, *p* = 0.087), gender (B = 0.17, SE = 0.13, β = 0.14, t = 1.35, *p* = 0.179), satisfaction with virtual assistants (B = 0.06, SE = 0.07, β = 0.09, t = 0.89, *p* = 0.375), satisfaction with route suggestions (B = −0.19, SE = 0.13, β = −0.16, t = −1.49, *p* = 0.140), satisfaction with facial recognition (B = 0.09, SE = 0.09, β = 0.10, t = 1.04, *p* = 0.299) and satisfaction with chatbots (B = 0.02, SE = 0.08, β = 0.03, t = 0.25, *p* = 0.801).

### 3.3. Perceptions Towards AI in Tele-Emergency Medicine

A total of 392 participants answered at least one of the three open-ended questions. The demographic characteristics of this group are comparable with the demographic characteristics of the main sample: 60.5% were women, the mean age was 41.41, 62% had a university degree, and 30.4% had a high school diploma as their highest level of education.

**Figure 6:**
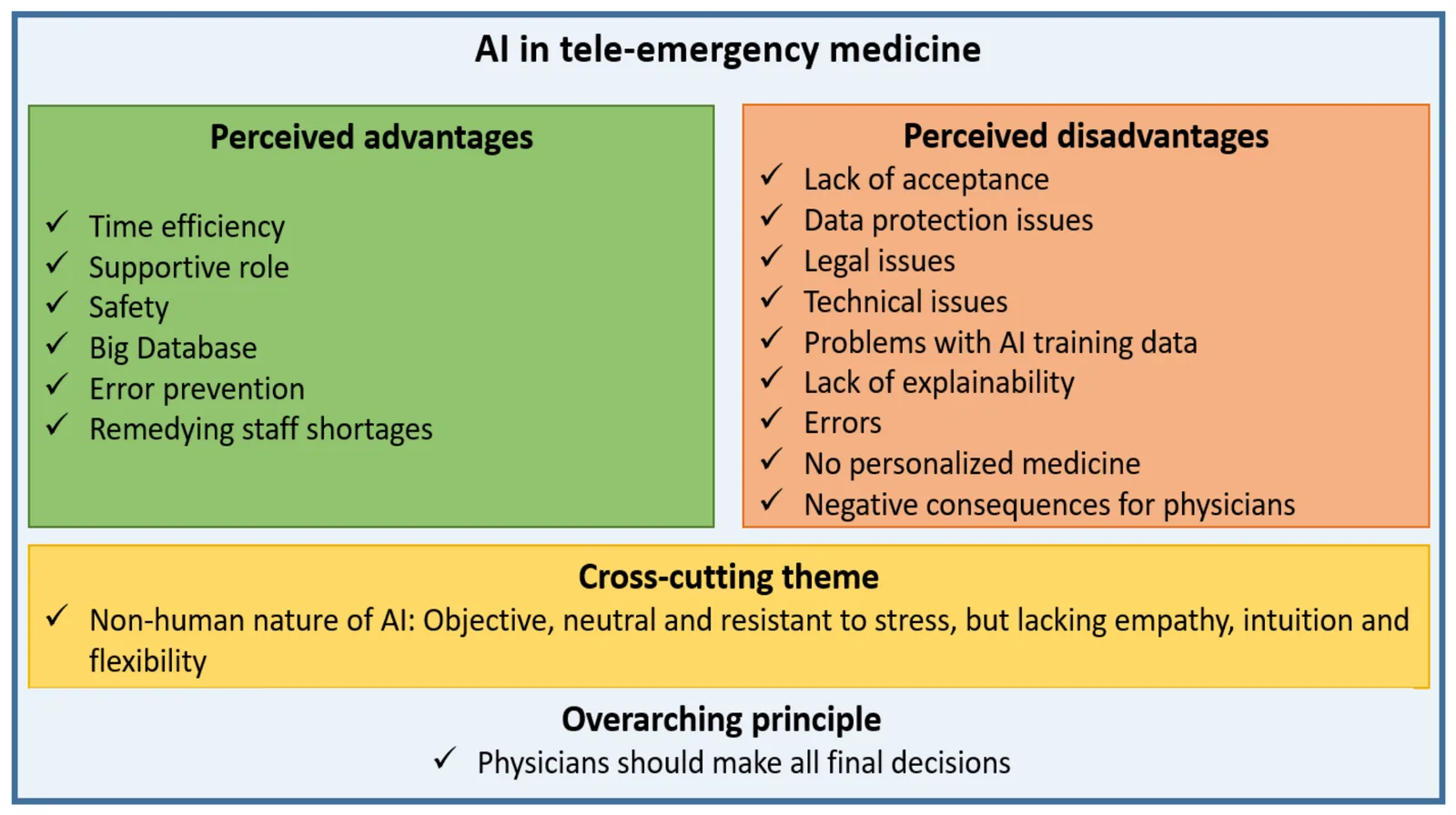
Categories reflecting the perceptions of AI-supported tele-emergency medicine (N = 392).

An inductive analysis [54] of the open-ended responses yielded several recurring categories reflecting participants’ perceptions of AI-supported tele-emergency medicine (Figure 6).

#### 3.3.1. Qualitative Findings: Perceived Advantages of AI in Tele-Emergency Medicine

- Time efficiency. Participants perceived AI as a means to accelerate workflows and enable faster responses in emergencies. AI was expected to rapidly identify critical indicators and thereby free up time for tele-emergency physicians to treat more patients, particularly in regions with limited physician availability. Another advantage mentioned was AI’s ability to provide rapid access to medical knowledge, which could facilitate faster decision-making.

*“As an advantage, I see that treatment can possibly be started more quickly.”*

*“Rapid availability of possible diagnoses and treatment measures, which serve as an overview for the emergency physician.”*

- Supportive role. AI was viewed as a “thinking” tool capable of providing systematic support for a range of tasks, including diagnostics (also for rare diseases), electrocardiogram and image analysis, and administrative tasks. Respondents compared AI to a medical colleague whom they would consult for professional advice. They highlighted its potential to support physicians in stressful situations and with patient documentation. Overall, participants believed that AI could facilitate the work of tele-emergency physicians and benefit the healthcare system.

*“Access to a larger database of clinical cases. Faster analysis of symptoms. Great help with rare diseases or diseases that are not typical for this geographical area.”*

*“Less experienced physicians could gain confidence and receive support as a result.”*

- Safety. Respondents perceived the AI system as a tool to enhance patient safety and improve care by providing diagnostic suggestions and warnings about drug interactions and contraindications. They expected AI to be familiar with clinical guidelines and procedural instructions, thereby helping physicians deliver guideline-compliant care. Participants noted that AI could improve safety not only for patients but also for medical staff by acting as a “second medical opinion”.

*“I associate this with the hope that, regardless of the individual qualifications of an emergency physician, AI can ensure comprehensive high-quality emergency care.”*

*“AI could be seen as a brief exchange with an experienced colleague, potentially reducing the likelihood of overlooking something.”*

- Big database. Responders considered AI as a means of expanding tele-emergency physicians’ expertise in the field of emergency medicine, as it is able to access and process more knowledge than any individual physician. This was seen as particularly helpful for identifying rare diseases and symptoms in unusual cases.

*“A physician, even an emergency physician, doesn’t always know everything, so I think AI can be very helpful in this regard, for example, in identifying or verifying rarer diagnoses more quickly.”*

*“An AI can theoretically gather and use all the existing knowledge in this field [the emergency medical field], which would be impossible for humans.”*

- Error prevention. Participants believed that AI could reduce the occurrence of errors and facilitate their rapid detection. They highlighted its potential to prevent tunnel vision, lapses in concentration, negligence errors, medication and treatment mistakes, misdiagnoses, and stress-related errors.

*“Lower error rate when physician and AI work together (four-eyes principle).”*

*“Errors/omissions are detected more quickly.”*

- Remedying staff shortages. Respondents suggested that AI could help remedy staff shortages or even (partially) serve as a substitute for tele-emergency physicians. They believed that AI could reduce costs and enable more efficient use of resources, thereby increasing the availability of tele-emergency physicians.

*“It is possible that the emergency physicians involved will be relieved in terms of time, allowing them to handle a higher number of emergencies.”*

*“Relief in the event of staff shortages, if necessary.”*

#### 3.3.2. Qualitative Findings: Perceived Disadvantages of AI in Tele-Emergency Medicine

- Lack of acceptance. Participants expressed concerns that both tele-emergency physicians and patients might not accept the use of AI. They noted that patients, especially older ones, might be skeptical or hesitant to trust AI-supported decisions. Participants assumed that this could negatively affect treatment outcomes and reduce patient adherence. They also suggested that AI could appear unfamiliar to physicians and patients, further limiting its acceptance.

*“The work of tele-emergency physicians tends to be viewed with doubt from skeptical people, because they have the feeling that they are being diagnosed by a ‘machine’ rather than a medical specialist. This could influence the patient’s compliance with their treatment.”*

*“People’s skepticism (especially the elderly).”*

- Data protection issues. Concerns were voiced regarding data protection. Participants feared that health data could be stolen, shared with third parties without patients’ consent, or otherwise misused. A recurring concern was that patients might have little or no control over their personal information.

*“It is extremely important to me that there is transparent communication about who has access to my health data, when they have it, and how it may be anonymized—and that no pharmaceutical lobbyists are given access to my data.”*

*“Handling of personal data, for example the sharing of data without the patient’s knowledge and without them having any control over what happens to it.”*

- Legal issues. Responders questioned the legal implications of errors involving AI and raised concerns about responsibility in such cases. They feared that tele-emergency physicians might have to justify decisions that diverge from AI recommendations and could feel pressured to follow AI suggestions for liability reasons. Participants criticized the current lack of adequate legal frameworks for AI use in (tele-)emergency medicine.

*“Who is liable if the AI makes a treatment error? The physician still needs to be able to understand what the AI is suggesting and should not blindly trust it.”*

*“The tele-emergency physician might rely more on AI than on their own experience (in order to protect themselves legally).”*

- Technical issues. Some participants questioned whether AI is currently sufficiently advanced to be reliably applied in emergency medical settings. Concerns were also raised that the technology could fail, particularly due to connection problems or server overload.

*“Interaction between different software—if it doesn’t run smoothly, it wastes unnecessary time.”*

*“(…) possible difficulties in areas with poor network coverage.”*

- Problems with AI training data. Respondents suspected that training data might be biased, which could result in discrimination against certain patient groups. They were concerned that AI might fail to recognize rare diseases or exceptional cases if these were not represented in the training dataset. Furthermore, participants assumed that AI’s decision-making capacity would be limited if the training datasets were too small or of unclear or poor quality.

*“Possible errors/misdiagnoses, which could, however, be reduced by extensive training of the AI.”*

*“Risk of bias: AI could make incorrect treatment recommendations for rare patient groups with rare diseases.”*

- Lack of explainability. Insufficient transparency in AI-generated suggestions was perceived as problematic. Participants argued that if tele-emergency physicians do not understand the underlying algorithms, they cannot fully grasp the rationale behind AI recommendations. This was perceived as particularly problematic when AI suggestions diverged from physicians’ assessments, as it could hinder physicians’ ability to evaluate them.

*“Too much trust in the statements made by the AI. Too little understanding of how the AI operates.”*

*“When information from the AI seems to be incorrect, it is difficult to understand how it [AI] arrived at that conclusion.”*

- Errors. Participants expressed concerns that AI could generate incorrect suggestions, leading to misguided decisions in tele-emergency medicine. Some of them emphasized that AI errors may be difficult to detect and could therefore remain unnoticed and uncorrected for extended periods. In addition to errors made by AI, participants also pointed out the risk of user-related errors, for instance, through incorrect handling of the system. Both faulty AI outputs and improper use could contribute to misdiagnoses, tunnel vision, and treatment errors.

*“Possibly errant AI or trusting AI too much.”*

*“Incorrect handling, poor communication, lack of competence, empathy and analytical skills.”*

- No personalized medicine. Some respondents objected that AI would merely provide “standard diagnoses” and recommend “standard treatments”. They believed that AI would mainly rely on common cases in emergency medicine and fail to adequately consider specific characteristics and needs of individual patients.

*“It may be that standard diagnoses are made rather than addressing the individual case.”*

*“Perhaps it [AI] suggests the things that occur most frequently first. Rare things may not be considered.”*

- Negative consequences for physicians. Participants raised concerns regarding deskilling and automation bias. They perceived a risk that AI could negatively affect physicians’ practical skills and expertise and that tele-emergency physicians might rely too heavily on AI and fail to critically evaluate its suggestions. Such overreliance could increase the risk of errors, hinder learning, and deteriorate physicians’ abilities and intuition. Participants further suggested that constant reliance on AI could foster a sense of helplessness in the event of a system failure and might diminish physicians’ sense of responsibility. Conversely, it was also hypothesized that AI could interfere with physicians’ work if it generates excessive or incorrect suggestions, leading to distractions and delays.

*“Eventually, a physician might rely too much on it [AI] and no longer trust enough their own abilities and experience (which could be problematic,* e.g., *if the technology fails), and the physician’s ‘gut feeling’ might also be lost.”*

*“This [AI] makes tele-emergency physicians lazy and causes them to lose skills.”*

*“Possibly too much trust in the system and uncritical acceptance of the proposed measures.”*

#### 3.3.3. Qualitative Findings: The Non-Human Nature of AI

Participants repeatedly referred to the non-human nature of AI and viewed it both as an advantage and as a disadvantage. From one perspective, participants noted that AI functions unaffected by stressful situations. Unlike physicians, AI was assumed to operate independently of time and fatigue, without overlooking information. Participants believed that AI outperforms humans in identifying patterns and correlations. Some participants emphasized that its neutrality—being free from prejudice, emotions, or personal biases—enables objective evaluation.

*“Less prone to errors; independent of the physician’s physical condition; suggests optimal follow-up care.”*

*“Artificial intelligence does not overlook rare diagnoses, can identify drug interactions, provide recommendations, and does not forget anything due to stress*.”

*“There are no prejudice, for example, based on sympathies.”*

From another perspective, AI was seen as lacking creativity, flexibility, and spontaneity—qualities considered valuable in the context of tele-emergency medicine. Participants argued that AI is unable to adequately interpret patients’ environments and lacks basic human intuition as well as “gut feeling”. Concerns were raised about AI’s inability to express empathy, resulting in a cold and impersonal approach to patient care.

*“AI cannot be creative—yet creativity is sometimes essential in emergency medical services (on-site).”*

*“The intuition and experiential intelligence of the tele-emergency physician cannot be simulated; AI cannot assess the environment, nor is it capable of empathy.”*

*“AI may not be able to contextualize the overall situation in the same way a physician can; an experienced physician may be able to reach a conclusion more quickly, based on their ‘gut feeling’ (…).”*

#### 3.3.4. Qualitative Findings: Physicians Should Make All Decisions

In their written answers, the participants consistently emphasized that tele-emergency physicians should make all final decisions during rescue operations and that AI should be used only as a supportive tool.

*“It is important to see AI only as a support tool and not as a decision-maker.”*

*“(…) I envision AI as a support tool and clinical decision support system; the decision regarding treatment should continue to be made by the tele-medical staff.”*

Respondents also stressed that tele-emergency physicians must maintain control over their work, critically evaluate AI suggestions, and remain constantly aware of their responsibilities.

*“The ‘human’ tele-emergency physician must always be aware that it is their responsibility to make and be accountable for the decision.”*

*“The AI must continue to be developed, but it must always remain controllable by humans.”*

## 4. Discussion

Participants generally expressed a moderate level of agreement with positively framed statements about AI-supported tele-emergency medicine. Acceptance was higher among men than among women and tended to increase with age. The finding that men showed greater acceptance is in line with earlier research on AI in healthcare [12]. The observed age effect may be partly explained by the online administration of the questionnaire. Participation required access to internet-enabled devices and sufficient digital literacy, meaning that older respondents were likely to represent older adults with higher levels of digital literacy and better access to digital technologies than the older population as a whole [56].

No significant differences in acceptance were found across different educational levels. However, the sociodemographic characteristics of the participants revealed difficulties in reaching individuals with an education level below a high school diploma, resulting in an underrepresentation of this group. This may have introduced selection bias, as educational attainment is associated with differences in health literacy [57] and knowledge about AI [58]. Consequently, the findings may not fully capture the perspectives of individuals with lower educational attainment.

As expected, people who use AI more often in their everyday lives showed greater acceptance of AI-supported tele-emergency medicine. Age and average use of route suggestions, facial recognition, and chatbots were found to be significant positive predictors for acceptance. Participants highlighted the importance of time efficiency, safety, accuracy of AI suggestions, and absence of errors, as well as the need for adequate training data, acceptance, and responsible system use, data security and legal safeguards, technical reliability, and positive impact on physicians’ work and patients’ outcomes. These findings align with the existing literature and reflect important bioethical principles. The use of a convenience sample, however, may have influenced the diversity of the participants included, potentially limiting the transferability of the findings [59].

The specific conditions of prehospital emergency care are important for interpreting these findings. Concerns regarding trust, erroneous recommendations, explainability, and responsibility may be particularly relevant in tele-emergency care, where opportunities to verify AI-generated recommendations or seek additional opinions may be limited [32]. Participants’ strong preference expressed for maintaining physician control supports the importance of meaningful human oversight in high-stakes medical decision-making [60]. Questions of responsibility, accountability, and the implementation of appropriate regulatory safeguards are imperative when integrating AI into emergency medical workflows [26].

The potential advantages and disadvantages identified by participants should also be considered in relation to the intended supportive role of the system in the specific prehospital setting. The AI-supported tele-emergency system has the potential to assist physicians in several ways, including suggesting possible diagnoses, treatment options, and options for further patient transportation. Such functions may reduce cognitive workload and support real-time decision-making [27]. However, these potential benefits of such an approach are dependent on the quality and reliability of the underlying system and data. Erroneous or biased recommendations as well as technical failures may introduce additional risks [61]. AI should therefore be regarded as a supportive tool rather than a replacement for physician judgment.

To address core ethical issues related to the acceptance of AI-supported tele-emergency medicine, the five MEESTAR dimensions (care, autonomy, safety, self-conception, and justice) for ethical evaluation [42,43] from the main part of the questionnaire are discussed separately in the following sections. The aim of this ethical reflection is to discuss the ethical issues related to the use of AI in the tele-emergency medical system, rather than to review the existing system, which has already been the focus of multiple scientific investigations, e.g., [25,62,63,64,65,66,67,68,69,70].

### 4.1. Care

For the purposes of the questionnaire, “care” was defined as the ability to care for others in situations in which they cannot care for themselves [44]. According to our results, participants believed that AI could help physicians make accurate diagnoses, prevent misdiagnosis, follow standard operating procedures more closely, improve treatment accuracy, and document patient cases more efficiently. These capabilities are frequently cited in the literature [71,72,73,74,75,76,77] as ways in which AI can enhance the quality of patient care [74,78] and reduce treatment errors [72,73,77], as AI is perceived to operate independently of human judgment and error [77]. From an ethical perspective, the potential to enhance diagnostic accuracy and prevent errors aligns with the classical bioethical principles of “beneficence” and “non-maleficence” [79]. Understood as “do only good” and “do no harm” [80], these two principles complement each other: on the one side, harm should be actively prevented, and on the other side, intentional harmful actions should be avoided [79]. This encompasses preserving patient privacy when using AI and avoiding its misuse [80].

In practical prehospital care, AI support should not delay established emergency treatment or stabilization measures and should ultimately contribute to positive patient outcomes. Its utilization should not result in harm to patients or other stakeholders, whether through effects on health, privacy, or other aspects of care.

### 4.2. Autonomy

“Autonomy” in the survey referred to the freedom of individuals to make their own decisions [44]. For patients, this means the right to make informed decisions about their health and treatment [81], which is possible only if they receive understandable information on how an AI system generates suggestions. For physicians, it concerns their freedom of decision-making [44] while performing their duties. Participants warned that physicians’ autonomy may be undermined by AI if they rely too heavily on it, especially if they assume that it always functions accurately or avoid disagreeing with AI-generated suggestions and fear legal consequences in the event of an error. These results are consistent with the existing literature [82,83,84] and suggest that physicians may lose training opportunities and professional skills as a consequence [61,83]. This, in turn, may lead to uncritical reliance on potentially erroneous systems as well as helplessness and potentially endangering patients’ lives in the case of breakdowns [61,85].

Physician autonomy can be supported by ensuring that AI recommendations remain advisory, understandable, and easily overridable. Meaningful human oversight requires that physicians be able to understand, question, and disagree with AI recommendations when they consider them inappropriate [60]. In accordance with the ethical principle of autonomy, patients should also be informed about the implementation of AI in their healthcare [86]. However, this may not be possible in many emergency situations due to the patients’ conditions and the urgency of the situation [87]. Therefore, communicative strategies should be applied to inform the population about the AI integration in tele-emergency medicine [61,88].

### 4.3. Safety

The concept of “safety” covered AI competence, therapy safety, and data security [43]. Our findings align with existing research indicating that erroneous AI suggestions may result from incomplete or inaccurate training data [89,90], which may lead to data bias [74] and stigmatization of certain patient groups [77]. Overreliance on AI may contribute to loss of professional skills [89], reduced engagement in continuing education among physicians [71], and increased dependence on technology [14]. There are concerns that AI may devalue the profession of physicians [72] or replace human physicians [75]. As AI lacks empathy [71,75], its implementation could lead to the dehumanization of healthcare [72,73,75]. Such outcomes risk violating the ethical principle of “beneficence”, which emphasizes not only providing the best possible treatment but also delivering compassionate, patient-centered care [79].

Similar to previous research, our study found that data protection and legal issues are frequent concerns regarding AI implementation in medical practice. AI is perceived as potentially compromising patient privacy [73,74,89] by risking improper data use [74,77]. Insufficiently regulated liability when working with AI [71], as well as a lack of transparency in the way it operates [89,91], are seen as significant disadvantages, particularly given that AI could make mistakes [74]. Such errors may lead to misdiagnoses or mistreatment when AI-generated information is not verified by the physician [77]. These are issues not only of “non-maleficence”, but also of “autonomy” [79].

From a practical standpoint, the concerns raised by participants regarding diagnostic and therapeutic errors, algorithmic bias, legal liability, and overreliance on AI underscore the importance of appropriate safeguards when AI is introduced into emergency practice. These safeguards should encompass measures addressing data security and the ongoing monitoring of AI-related risks, as well as clear responsibilities and continued multidisciplinary oversight [92].

### 4.4. Self-Conception

“Self-conception” was understood as the perception of potential changes, including those in professional roles due to technology [45]. Participants stressed that physicians must retain ultimate authority over treatment decisions to avoid a perceived loss of humanity in care. They called for a legally binding framework that guarantees both patient safety and clear responsibilities for medical staff. To prevent potential perceived dehumanization, it is important that physicians retain the authority to make final treatment decisions [71,76,93]. It is crucial to create a legally binding framework for the implementation of AI in healthcare [61,85] and, more specifically, in tele-emergency medicine to guarantee legal safeguards for medical professionals, as well as to ensure patient safety in the context of optimal medical care and secure handling of health and personal data, and to provide legal clarity regarding physicians’ roles, rights, and responsibilities [61]. Explainability of how AI systems operate and handle patient data was seen as vital for acceptance and for building trust among physicians and patients [94,95]. Therefore, in order to ensure acceptance of the new technology, the population must be carefully and thoroughly informed about its implementation and characteristics [61]. This includes providing understandable information about its potential, limitations, and risks, thereby promoting transparent dialog and informed decision-making [61,85,94]. In parallel, it is important to educate users about the correct and responsible use of AI so that they are aware of its possible advantages and disadvantages and of their specific role in this interaction [61,96]. Therefore, continuing education is recommended to prepare tele-emergency physicians to appropriately use AI systems and critically evaluate their recommendations [97].

### 4.5. Justice

“Justice” was defined as fairness and the absence of detriment to the tele-emergency physician [46]. In both our study and the existing literature, AI is assumed to be capable of reducing workload [71,74,89,93] due to its faster and more precise data processing compared to humans [89]. Moreover, AI is able to collect and analyze large datasets [14,75]. This is believed to enable physicians to invest more time in other tasks requiring their full, undivided attention [73,89]. Ethically, the use of medical AI is intended to benefit every individual involved, including physician users [98]. This aligns with the bioethical principles of “beneficence” and “justice” [79], which imply that physicians should work for the benefit of patients and in their best interests [99] and treat all patients equally, unless evidence suggests that specific circumstances warrant a different approach [100]. This encompasses a fair, equitable, and appropriate distribution of healthcare services, regardless of characteristics such as sex and gender, ethnicity, or social status, which could otherwise serve as a basis for discrimination [79].

To ensure equitable care, the performance of AI systems should be continuously monitored across relevant patient groups, rather than solely evaluating overall accuracy, because unrepresentative data can lead to clinically relevant disparities in system performance between subgroups [101].

The five MEESTAR dimensions reveal a balanced perspective that includes both hopes for better care, safety, and fairness as well as concerns about errors, reduced autonomy of patients and physicians, and risks to data privacy. Therefore, responsible implementation of AI in tele-emergency medicine requires robust and explainable algorithms, transparent communication about data handling, clear liability and regulatory frameworks, and dedicated educational programs for medical professionals and the public.

### 4.6. Limitations

The primary limitation of this study is the bias resulting from the sample selection. The sample was recruited using convenience methods and consisted of individuals with a high level of education. The online survey format and recruitment via institutional websites, social media, the website of a popular science magazine, and a patient organization mailing list may have primarily reached individuals with greater digital access, digital literacy, an interest in health and science, and familiarity with technology [102,103]. These factors limit the representativeness of the sample [102].

The characteristics of individuals who were more likely to participate may have affected both the overall acceptance levels and the observed differences between subgroups. Consequently, the findings should not be generalized to the entire German population. Future studies should employ more representative recruitment strategies and specifically include a diverse group of individuals with varying levels of education.

In addition, the findings are based on self-reported attitudes. Participants evaluated the system based on a written description rather than seeing or even experiencing it. Reported acceptance should therefore be interpreted as stated willingness rather than actual behavior, which may differ under the stress and uncertainty of a real emergency situation [104].

## 5. Conclusions

This study presents key ethical considerations surrounding the use of AI in tele-emergency medicine and reveals moderate acceptance of AI in this context. Associations were observed with gender, age, and the use of AI in everyday life. By exploring public perspectives on the use of AI in tele-emergency medicine, this study addresses an important gap in the literature. Participants expressed a balanced view, recognizing both potential benefits and possible risks. Specifically, they emphasized opportunities to enhance efficiency, safety, and diagnostic accuracy while also voicing concerns about reliability, accountability, data protection, and broader ethical implications.

Our findings align with existing research on AI in other areas of healthcare and resonate with established bioethical principles. As such, our findings provide a valuable foundation for the responsible and ethical integration of AI into tele-emergency medicine. Since the questionnaire was designed for the German population, further studies in other language areas and with different population groups would be beneficial for identifying potential sociocultural differences in acceptance.

## Figures and Tables

**Table 1:** Sociodemographic characteristics of the study sample (N = 497).

		N (%)
Gender	Female	301 (60.6%)
	Male	191 (38.4%)
	Other	4 (0.8%)
	Missing	1 (0.2%)
Highest level of education	University degree (German: Universitäts-/Fachhochschulabschluss)	295 (59.4%)
	High school diploma (German: Abitur/Fachhochschulreife)	149 (30.0%)
	Certificate of secondary education (German: Realschulabschluss/Mittlere Reife)	49 (9.9%)
	Certificate of basic education (German: Haupt-/Volkschulabschluss)	2 (0.4%)
	Other school qualification	2 (0.4%)
Age	M	41.46
	SD	14.44
	Range	18–83
Experience with emergency care	Yes	254 (51.1%)
	No	160 (32.2%)
	Missing	83 (16.7%)

**Table 2:** Spearman correlations between AI-system use/satisfaction and acceptance of AI-supported tele-emergency medicine.

	Use (ρ)	*p*	N	Satisfaction (ρ)	*p*	N
Virtual assistants	0.15 **	0.010	312	0.14	0.052	197
Route suggestions	0.23 **	<0.001	312	0.12 *	0.030	306
Facial recognition	0.18 **	0.001	308	0.08	0.242	194
Chatbots	0.24 **	<0.001	307	0.11	0.122	198

* Correlation significance level 0.05 (two-tailed); ** Correlation significance level 0.01 (two-tailed).

## Data Availability

The data presented in this study are available on request from the corresponding author due to ethical and data protection restrictions related to research involving human participants.

## Supplementary Materials

The following supporting information can be downloaded at: https://www.mdpi.com/article/10.3390/healthcare14172753/s1, File S1: Daily AI use and satisfaction items; File S2: Acceptance Scale.

## Abbreviations

The following abbreviations are used in this manuscript:

AI	Artificial intelligence
KIT^2^	KI-unterstützter Telenotarzt, English: AI-supported tele-emergency physician
MEESTAR	Model for Ethical Evaluation of Socio-Technical Arrangements
SPSS	Statistical Package for the Social Sciences
TAM	Technology Acceptance Model
UTAUT	Unified Theory of Acceptance and Use of Technology

## References

[1] Kennedy B., Tyson A., Saks E. Public Awareness of Artificial Intelligence in Everyday Activities: Limited enthusiasm in U.S. Over AI’s Growing Influence in Daily Life. United States: Pew Research Center 2023. https://www.pewresearch.org/?p=104383.

[2] Prandner D., Hagedorn L., Schmid U., Winter S., Woltran S. (2026). Are They Aware When AI is Used? And What do They Think that AI Should be used for?—Insights into the Digital Skills Austria III Study. Digital Humanism: First Interdisciplinary Science and Research Conference, DIGHUM 2025.

[3] Mohajer-Bastami A., Moin S., Ahmad S., Ahmed A.R., Pouwels S., Hajibandeh S., Yang W., Parmar C., Kermansaravi M., Khalil M. (2025). Artificial intelligence in healthcare: Applications, challenges, and future directions. A narrative review informed by international, multidisciplinary expertise. Front. Digit. Health.

[4] Rasche C., von Reinersdorff C.B., von Reinersdorff A.B., Reinecke A., Pfannstiel M.A. (2025). Erfolgreiche Patientenreise durch Künstliche Intelligenz [Successful patient journey through artificial intelligence]. Künstliche Intelligenz im Einsatz für die Erfolgreiche Patientenreise: Innovation, Integration und Stärkung.

[5] Stallkamp J., Horsch J., Karstensen L., Pfannstiel M.A. (2022). KI-Systeme für die nächste Medizintechnikgeneration [AI systems for the next generation of medical technology]. Künstliche Intelligenz im Gesundheitswesen: Entwicklungen, Beispiele und Perspektiven.

[6] Ouanes K., Farhah N. (2024). Effectiveness of Artificial Intelligence (AI) in Clinical Decision Support Systems and Care Delivery. J. Med. Syst..

[7] Adams S.J., Tang R., Babyn P. (2020). Patient Perspectives and Priorities Regarding Artificial Intelligence in Radiology: Opportunities for Patient-Centered Radiology. J. Am. Coll. Radiol..

[8] Daniyal M., Qureshi M., Marzo R., Aljuaid M., Shahid D. (2024). Exploring clinical specialists’ perspectives on the future role of AI: Evaluating replacement perceptions, benefits, and drawbacks. BMC Health Serv. Res..

[9] Schulte A., Suarez-Ibarrola R., Wegen D., Pohlmann P.F., Petersen E., Miernik A. (2020). Automatic speech recognition in the operating room—An essential contemporary tool or a redundant gadget? A survey evaluation among physicians in form of a qualitative study. Ann. Med. Surg..

[10] Aras S., Drakos C., Manimangalam V., Nazir M.A., Bruns C., Smith D., Equils O. (2026). Influencing public acceptance of artificial intelligence (AI) in healthcare delivery. Front. Digit. Health.

[11] Baldus S.G., Wiesmann M., Habel U., Gerhards A., Hasan D., Weyland C.S., Truhn D., Hasl M.M., Clemes B., Nikoubashman O. (2026). Patients’ views on the use of artificial intelligence in healthcare: Artificial Intelligence Survey Aachen (AISA)—A prospective survey. Insights Imaging.

[12] Shah P., Mishra D., Shanmugam M., Vighnesh M., Jayaraj H. (2022). Acceptability of artificial intelligence-based retina screening in general population. Indian J. Ophthalmol..

[13] Tamori H., Yamashina H., Mukai M., Morii Y., Suzuki T., Ogasawara K. (2022). Acceptance of the Use of Artificial Intelligence in Medicine Among Japan’s Doctors and the Public: A Questionnaire Survey. JMIR Hum. Factors.

[14] Maassen O., Fritsch S., Palm J., Deffge S., Kunze J., Marx G., Riedel M., Schuppert A., Bickenbach J. (2021). Future Medical Artificial Intelligence Application Requirements and Expectations of Physicians in German University Hospitals: Web-Based Survey. J. Med. Internet Res..

[15] Kauttonen J., Rousi R., Alamäki A. (2025). Trust and Acceptance Challenges in the Adoption of AI Applications in Health Care: Quantitative Survey Analysis. J. Med. Internet Res..

[16] Levai C.M., Nussbaum L.A., Popa D., Burtic S., Căpăstraru B.F., Jugănaru I. (2025). Patient Perspectives on AI- and XR-Enabled Telemedicine: A Cross-Sectional Survey in Romania. Healthcare.

[17] Aljamaan F., Malki K.H., Alhasan K., Jamal A., Altamimi I., Khayat A., Alhaboob A., Abdulmajeed N., Alshahrani F.S., Saad K. (2024). ChatGPT-3.5 System Usability Scale early assessment among Healthcare Workers: Horizons of adoption in medical practice. Heliyon.

[18] Busch F., Hoffmann L., Xu L., Zhang L.J., Hu B., García-Juárez I., Toapanta-Yanchapaxi L.N., Gorelik N., Gorelik V., Rodriguez-Granillo G.A. (2025). Multinational Attitudes Toward AI in Health Care and Diagnostics Among Hospital Patients. JAMA Netw. Open.

[19] Gundlack J., Negash S., Thiel C., Buch C., Schildmann J., Unverzagt S., Mikolajczyk R., Frese T. (2025). PEAK consortium Artificial Intelligence in Medical Care—Patients’ Perceptions on Caregiving Relationships and Ethics: A Qualitative Study. Health Expect..

[20] Chen J., Wang H., Qiu X. (2026). Who is more trustworthy? The influence mechanism of AI vs. human doctor triage on user trust: Testing the mediating effect of psychological distance, and a multiple moderating effects analysis. Front. Psychol..

[21] Bruni T., Heinrichs B., Pfannstiel M.A. (2025). Künstliche Intelligenz in der Behandlung von Diabetes bei minderjährigen Patienten—Ethische Aspekte [Artificial intelligence in the treatment of diabetes in underage patients—Ethical aspects]. Künstliche Intelligenz im Einsatz für die Erfolgreiche Patientenreise: Innovation, Integration und Stärkung.

[22] Giebel G.D., Raszke P., Nowak H., Palmowski L., Adamzik M., Heinz P., Tokic M., Timmesfeld N., Brunkhorst F.M., Wasem J. (2025). Improving AI-Based Clinical Decision Su port Systems and Their Integration Into Care from the Perspective of Experts: Interview Study Among Different Stakeholders. JMIR Med. Inform..

[23] Labkoff S., Oladimeji B., Kannry J., Solomonides A., Leftwich R., Koski E., Joseph A.L., Lopez-Gonzalez M., A Fleisher L., Nolen K. (2024). Toward a responsible future: Recommendations for AI-enabled clinical decision support. J. Am. Med. Inform. Assoc..

[24] Bundesärztekammer [German Medical Association] (2025). Von Ärztlicher Kunst mit Künstlicher Intelligenz [From Medical Art to Artificial Intelligence].

[25] Schröder H., Beckers S.K., Borgs C., Sommer A., Rossaint R., Grüßer L., Felzen M. (2024). Long-term effects of a prehospital telemedicine system on structural and process quality indicators of an emergency medical service. Sci. Rep..

[26] Machado C., Hessel S. (2026). Artificial intelligence in the prehospital setting—Potentials, challenges, and practice-relevant fields of application in emergency medical services. BMC Artif. Intell..

[27] Bai C., Zhang Z., Xu Y., Luo X., Adelgais K. (2025). Enhancing prehospital decision-making: Exploring user needs and design considerations for clinical decision support systems. BMC Med. Inform. Decis. Mak..

[28] World Health Organization (2025). Prehospital Emergency Care—Operational Guidance for Ambulance Systems.

[29] Amiot F., Potier B. (2025). Artificial Intelligence (AI) and Emergency Medicine: Balancing Opportunities and Challenges. JMIR Med. Inform..

[30] Petrella M.J. (2024). The AI Future of Emergency Medicine. Ann. Emerg. Med..

[31] Pfannstiel M.A., Pfannstiel M.A. (2025). Künstliche Intelligenz im Einsatz für die erfolgreiche Patientenreise” [Introduction “Artificial Intelligence in use for a successful patient journey”]. Künstliche Intelligenz im Einsatz für die Erfolgreiche Patientenreise: Innovation, Integration und Stärkung.

[32] Durdova N., Groß D., Schmidt M., Schröder H., Felzen M., Irrgang M., Wilhelmy S. (2026). AI support in prehospital telemedicine: Perspectives of tele-emergency physicians and ethical considerations. Digit. Health.

[33] Leiner D.J. (2023). SoSci Survey [Computer Software].

[34] Schmidt M., Wilhelmy S., Czaplik M., Kowark P., Inthorn J., Seising R. (2021). Akzeptanz und ethische Implikationen der Ärztlichen Telekonsultation. Das Beispiel Schmerztherapie [Acceptance and Ethical Implications of Physician Teleconsultation: The Case of Pain Therapy]. Digitale Patientenversorgung: Zur Computerisierung von Diagnostik, Therapie und Pflege.

[35] Adell E., Varhelyi A., Nilsson L., Horberry T., Regan M.A., Stevens A. (2014). The Definition of Acceptance and Acceptability. Driver Acceptance of New Technology—Theory, Measurement and Optimisation.

[36] AlQudah A.A., Al-Emran M., Shaalan K. (2021). Technology Acceptance in Healthcare: A Systematic Review. Appl. Sci..

[37] Nadal C., Sas C., Doherty G. (2020). Technology Acceptance in Mobile Health: Scoping Review of Definitions, Models, and Measurement. J. Med. Internet Res..

[38] Scheuer D. (2020). Akzeptanz von Künstlicher Intelligenz—Grundlagen Intelligenter KI-Assistenten und deren Vertrauensvolle Nutzung.

[39] Amisha, Malik P., Pathania M., Rathaur V.K. (2019). Overview of artificial intelligence in medicine. J. Fam. Med. Prim. Care.

[40] Naß E., Renno C., Rörtgen D., Skorning M. (2010). Forschungsprojekt Med-on@ix: Telemedizin im Rettungswesen [Research Project Med-on@ix: Telemedicine in Emergency Medical Services]. Dtsch. Arztebl..

[41] Fröhl U., Friedrich C. (2022). Quick Guide Onlinefragebogen—Wie Sie Ihre Zielgruppe Professionell im Web Befragen [Quick Guide Online Questionnaire—How to Professionally Survey Your Target Group Online].

[42] Manzeschke A., Weber K., Frommeld D., Manzeschke A., Fangerau H. (2015). MEESTAR: Ein Model angewandter Ethik im Bereich assistiver Technologien [MEESTAR: A model of applied ethics in assistive technology]. Technisierung des Alltags. Beitrag für ein Gutes Leben?.

[43] Manzeschke A., Weber K., Rother E., Fangerau H. (2013). Ethische Fragen im Bereich Altersgerechter Assistenzsysteme: Ergebnisse der Studie [Ethical Issues in the Field of Age-Appropriate Assistive Systems: Study Results].

[44] Fink V., Börner A., Eibl M. Living-Lab and Experimental Workshops for Design of I-RobEka Assistive Shopping Robot: ELSI Aspects with MEESTAR. Proceedings of the 29th IEEE International Conference on Robot and Human Interactive Communication (RO-MAN).

[45] Weber K. MEESTAR2—Ein erweitertes Modell zur ethischen Evaluierung soziotechnischer Arrangements [MEESTAR2—An expanded model for the ethical evaluation of socio-technical arrangements]. Proceedings of the Zweite Transdisziplinäre Konferenz zum Thema “Technische Unterstützungssysteme, die die Menschen Wirklich Wollen”.

[46] Bartneck C., Lütge C., Wagner A.R., Welsh S. (2019). Ethik in KI und Robotik [Ethics in AI and Robotics].

[47] Davis F.D. (1989). Perceived Usefulness, Perceived Ease of Use, and User Acceptance of Information Technology. MIS Q..

[48] Venkatesh V., Morris M.G., Davis G.B., Davis F.D. (2003). User Acceptance of Information Technology: Toward A Unified View. MIS Q..

[49] Meade A., Craig B. (2012). Identifying Careless Responses in Survey Data. Psychol. Methods.

[50] Roßmann J. Data quality in web surveys of the German longitudinal election study 2009. Proceedings of the Paper Presented at the 3rd ECPR Graduate Conference.

[51] Lazdauskas T., McDevitt S.C. (2025). Careless Responding May Reduce Data Quality in Self-Report Questionnaire Research: Evidence from Adult Temperament Samples from Two Cultures. Psychol. Rep..

[52] Field A. (2016). Discovering Statistics Using IBM SPSS Statistics.

[53] IBM Corp (2023). IBM SPSS Statistics for Windows.

[54] Mayring P. (2022). Qualitative Inhaltsanalyse—Grundlagen und Techniken [Qualitative Content Analysis—Principles and Techniques].

[55] George D., Mallery P. (2016). IBM SPSS Statistics 23 Step by Step: A Simple Guide and Reference.

[56] Khan S., Webster S., Puxty J., Robertson M. (2026). Key Challenges and Barriers to Digital Literacy for Older Adults: Scoping Review. JMIR Aging.

[57] Simpson R.M., Knowles E., O’Cathain A. (2020). Health literacy levels of British adults: A cross-sectional survey using two domains of the Health Literacy Questionnaire (HLQ). BMC Public Health.

[58] Gillespie N., Lockey S., Ward T., Macdade A., Hassed G. (2025). Trust, Attitudes and Use of Artificial Intelligence: A Global Study 2025. Univ. Melb..

[59] Golzar J., Noor S., Tajik O. (2022). Convenience Sampling. Int. J. Educ. Lang. Stud..

[60] van de Sande D., Economou-Zavlanos N., van Genderen M.E. (2026). Meaningful oversight of medical AI beyond human in the loop. npj Digit. Med..

[61] Deutscher Ethikrat [German Ethics Council] (2023). Mensch und Maschine—Herausforderungen Durch Künstliche Intelligenz [Humans and Machines—Challenges of Artificial Intelligence].

[62] Schröder H., Beckers S.K., Borgs C., Rossaint R., Felzen M. (2023). Update telenotfallmedizin: Status quo und Ausblick [Update tele-emergency medicine: Status quo and perspectives]. Anaesthesiologie.

[63] Beierle A., Beierle S., Pitsch M., Panagiotidis D., Larmann J., Beckers S.K., Felzen M., Schröder H. (2025). Telemedicine for prehospital respiratory emergencies in a retrospective quality analysis. Sci. Rep..

[64] Bergrath S., Czaplik M., Rossaint R., Hirsch F., Beckers S.K., Valentin B., Wielpütz D., Schneiders M.T., Brokmann J.C. (2013). Implementation phase of a multicentre prehospital telemedicine system to support paramedics: Feasibility and possible limitations. Scand. J. Trauma. Resusc. Emerg. Med..

[65] Felzen M., Beckers S.K., Kork F., Hirsch F., Bergrath S., Sommer A., Brokmann J.C., Czaplik M., Rossaint R. (2019). Utilization, safety, and technical performance of a telemedicine system for prehospital emergency care: Observational study. J. Med. Internet Res..

[66] Hess P.P., Czaplik M., Hess J., Schröder H., Beckers S.K., Follmann A., Pitsch M., Felzen M. (2025). Comparison of the diagnostic concordance of tele-EMS and EMS physicians in the emergency medical service—A subanalysis of the TEMS-trial. Front. Digit. Health.

[67] Katzenmeier C., Schrag-Slavu S. (2021). Telenotarzt: Berufsrecht, Haftungsrecht, Medizinprodukterecht, Datenschutzrecht [Tele-Emergency Physician: Professional Law, Liability Law, Medical Device Law, Data Protection Law].

[68] Kuntosch J., Metelmann B., Zänger M., Maslo L., Fleßa S. (2021). Das telenotarzt-system als Innovation im Rettungsdienst: Potenzialbewertung durch Mitarbeiter deutscher Einsatzleitstellen [The tele-emergency physician system as an innovation in the emergency medical service: Evaluation of potentials by employees of German ambulance control centres]. Gesunndheitswesen.

[69] Metelmann C., Metelmann B., Bartels J., Laslo T., Fleßa S., Hasebrook J., Hahnenkamp K., Brinkrolf P. (2019). Was erwarten Mitarbeiter der Notfallmedizin vom Telenotarzt? Ergebnisse einer Befragungsstudie vor der Einführung eines Telenotarztes in Vorpommern-Greifswald [Expectations of emergency personnel concerning a tele-emergency doctor. Results of a survey preceding the implementation of a telemedical system in a German emergency medical service]. Notf. Rettungsmed.

[70] Schröder H., Bouché L., Rehbock C., Beckers S., Felzen M. (2026). Supervision von Notärzten im Aachener Rettungsdienst durch den Telenotarzt [Supervision of emergency physicians in the Aachen rescue services by the tele-emergency physician]. Telenotfallmedizin.

[71] Ayad N., Schwendicke F., Krois J., van den Bosch S., Bergé S., Bohner L., Hanisch M., Vinayahalingam S. (2023). Patients’ perspectives on the use of artificial intelligence in dentistry: A regional survey. Head. Face Med..

[72] Jackson P., Ponath Sukumaran G., Babu C., Tony M.C., Jack D.S., Reshma V.R., Davis D., Kurian N., John A. (2024). Artificial intelligence in medical education—Perception among medical students. BMC Med. Educ..

[73] Pedro A.R., Dias M.B., Laranjo L., Cunha A.S., Cordeiro J.V. (2023). Artificial intelligence in medicine: A comprehensive survey of medical doctor’s perspectives in Portugal. PLoS ONE.

[74] Sahoo R.K., Sahoo K.C., Negi S., Baliarsingh S.K., Panda B., Pati S. (2025). Health professionals’ perspektives on the use of Artificial Intelligence in healthcare: A systematic review. Patient Educ. Couns..

[75] Salomon I., Olivier S. (2024). Artificial intelligence in medicine: Advantages and disadvantages for today and the future. Int. J. Surg. Open.

[76] Uihlein A., Beissel L., Ajlani A.H., Orzechowski M., Leinert C., Kocar T.D., Pankratz C., Schuetze K., Gebhard F., Steger F. (2024). Expectations and Requirements of Surgical Staff for an AI-Supported Clinical Decision Support System for Older Patients: Qualitative Study. JMIR Aging.

[77] Wurtz H.M., Manchester M., Valteau T., Hanson H., Scipion C., Federman A., Arias J.J. (2025). Artificial intelligence as a tool for cognitive impairment screening: Patient perspectives about benefits and limitations. Alzheimers Dement.

[78] van der Zander Q.E.W., van der Ende van Loon M.C.M., Janssen J.M.M., Winkens B., van der Sommen F., Masclee A.d.A.M., Schoon E.J. (2022). Artificial intelligence in (gastrointestinal) healthcare: Patients’ and physicians’ perspectives. Sci. Rep..

[79] Beauchamp T.L., Childress J.F. (2009). Principals of Biomedical Ethics.

[80] Floridi L., Cowls J., Beltrametti M., Chatila R., Chazerand P., Dignum V., Luetge C., Madelin R., Pagallo U., Rossi F. (2018). AI4People—An Ethical Framework for a Good AI Society: Opportunities, Risks, Principles, and Recommendations. Minds Mach..

[81] Marckmann G., Marckmann G. (2015). Grundlagen ethischer Entscheidungsfindung in der Medizin [Foundations of ethical decision-making in medicine]. Praxisbuch Ethik in der Medizin.

[82] Jones-Jang S.M., Park Y.J. (2022). How do people react to AI failure? Automation bias, algorithmic aversion, and perceived controllability. J. Comput. Mediat. Commun..

[83] Liedtke W., Langanke M. (2021). Der Einsatz von IT-basierten Decision-Support-Systemen in der medizinischen Versorgung aus verantwortungsethischer Sicht [The use of IT-based decision support systems in medical care—Perspectives from an ethics of responsibility]. Z. Med. Ethik..

[84] Murphy K., Ruggiero E.D., Upshur R., Willison D.J., Malhorta N., Cai C.J., Malhorta N., Lui V., Gibson J. (2021). Artificial intelligence for good health: A scoping review of the ethics literature. BMC Med. Ethics.

[85] World Health Organization (2021). Ethics and Governance of Artificial Intelligence for Health: WHO Guidance.

[86] Park H.J. (2024). Patient perspectives on informed consent for medical AI: A web-based experiment. Digit. Health.

[87] Trzeczak S. (2014). AG”Ethik in der Notfall-und Akutmedizin” der DGINA und der AEM. Das medizinisch-ethische Dilemma von Reanimationsentscheidungen bei Notfallpatienten [The medical–ethical dilemma regarding resuscitation decisions in emergency patients]. Notf. Rettungsmed..

[88] Rehbock C., Durdova N., Beckers S., Borgs C., Schröder H., Pfannstiel M.A. (2025). Einsatzszenarien und Potenziale von KI im Bereich der prähospitalen Telenotfallmedizin [Implementation scenarios and potential of AI in the field of prehospital tele-emergency medicine]. Künstliche Intelligenz im Einsatz Für die Erfolgreiche Patientenreise: Innovation, Integration und Stärkung.

[89] Parchmann N., Hansen D., Orzechowski M., Steger F. (2024). An ethical assessment of professional opinions on concerns, chances, and limitations of the implementation of an artificial intelligence-based technology into the geriatric patient treatment and continuity of care. Geroscience.

[90] Abdelwanis M., Alarafati H.K., Tammam M.M.S., Simsekler M.C.E. (2024). Exploring the risks of automation bias in healthcare artificial intelligence applications: A Bowtie analysis. J. Saf. Sci. Resil..

[91] Garcia-Gomez J.M., Blanes-Selva V., Romero C.A., de Bartolome Cenzano J.C., Mesquita F.P., Pazos A., Donate-Martinez A. (2025). Mitigating patient harm risks: A proposal of requirements for Ai in healthcare. Artif. Intell. Med..

[92] Sittig D.F., Singh H. (2025). Recommendations to Ensure Safety of AI in Real-World Clinical Care. JAMA.

[93] Fritsch S.J., Blankenhei A., Wahl A., Hetfeld P., Maassen O., Deffge S., Kunze J., Rossaint R., Riedel M., Marx G. (2022). Attitudes and perception of artificial intelligence in healthcare: A cross-sectional survey among patients. Digit Health.

[94] High-Level Expert Group on Artificial Intelligence (2019). Ethics Guidelines for Trustworthy AI.

[95] Liu Y., Liu C., Zheng J., Xu C., Wang D. (2025). Improving Explainability and Integrability of Medical AI to Promote Health Care Professional Acceptance and Use: Mixed Systematic Review. J. Med. Internet Res..

[96] Solanki P., Grundy J., Hussain W. (2023). Operationalising ethics in artifcial intelligence for healthcare: A framework for AI developers. AI Ethics.

[97] Schuitmaker L., Drogt J., Benders M., Jongsma K. (2025). Physicians’ required competencies in AI-assisted clinical settings: A systematic review. Br. Med. Bull..

[98] Swoboda W., Schmieder M., Fotteler M., Waibel A., Schobel J., Holl F., Lux T., Köberlein-Neu J., Müller-Mielitz S. (2022). Berücksichtigung der medizinischen Prinzipienethik bei der Evaluation von eHealth-Anwendungen. E-Health-Ökonomie II: Evaluation und Implementierung.

[99] Bester J.C. (2020). Beneficence, Interests, and Wellbeing in Medicine: What It Means to Provide Benefit to Patients. Am. J. Bioeth..

[100] Nash R.R., Wells M.J., Mazur K.A., Berg S.L. (2020). Ethical Principles in the Practice of Medicine. Ethical Issues in Pediatric Hematology/Oncology.

[101] Yang Y., Zhang H., Gichoya J.W., Katabi D., Ghassemi M. (2024). The limits of fair medical imaging AI in real-world generalization. Nat. Med..

[102] Szałata K., Czekalska M., Łabuzińska L., Bukowiec M., Worosz W., Sikorska L.M., Szpila G., Ćwil A. (2025). Bias in Online Health Surveys: Identifying and Overcoming Challenges. E-Methodology.

[103] Sedlár M., Grežo M. (2026). Willingness to participate in medical research: Unraveling the roles of scientific literacy and beliefs about medical researchers. Public Underst. Sci..

[104] Starcke K., Brand M. (2012). Decision making under stress: A selective review. Neurosci. Biobehav. Rev..

