# Supplementary material for: Artificial Intelligence in Prehospital Tele-Emergency Medicine: A Survey of Acceptance and Attitudes
Source: Healthcare (Basel). 2026 Aug 29;14(17):2753. doi: 10.3390/healthcare14172753 (PMC13565674; doi:10.3390/healthcare14172753)
Supplement: Supplementary file 1 [file healthcare-14-02753-s001.zip › healthcare-4497358-supplementary.pdf]

Supplement File S1. Daily AI use and satisfaction items

This supplement presents the items assessing participants' frequency of daily AI systems use and their satisfaction with each system. Four systems were included in the questionnaire. Both the originally used German items and the English translation are provided.

AI use, German original

Instruktion: Geben Sie bitte an, welche der aufgeführten KI-Systeme Sie nutzen und wie oft.

	täglich	mehrmals pro Woche	mehrmals pro Monat	mehrmals pro Jahr	nie	keine Angabe
**Sprachassistenten** (z.B. Alexa, Google Assistant, Siri)						
**Routenvorschläge** (z.B. Google Maps)						
**Gesichtserkennung** (z.B. auf Fotos oder zur Entsperrung des Smartphones)						
**Chatbots** (z.B. ChatGPT, Freddy Freshbot von Hello Fresh, Amazon-Chat-Support)						

AI use, English translation

Instruction: Please indicate which of the listed AI systems you use and how frequently.

	daily	several times a week	several times a month	several times a year	never	no response
**Virtual assistants** (e.g. Alexa, Google Assistant, Siri)						
**Route suggestions** (e.g. Google Maps)						
**Facial recognition** (e.g. on photos or for unlocking the smartphone)						
**Chatbots** (e.g. ChatGPT, Fredy Freshbot from Hello Fresh, Amazon-Chat-Support)						

AI satisfaction, German original

<u>Instruktion:</u> Geben Sie bitte an, wie zufrieden Sie mit der Nutzung der aufgeführten KI-Systeme sind.

	zufrieden	eher zufrieden	eher unzufrieden	unzufrieden	noch nie benutzt	keine Angabe
**Sprachassistenten** (z.B. Alexa, Google Assistant, Siri)						
**Routenvorschläge** (z.B. Google Maps)						
**Gesichtserkennung** (z.B. auf Fotos oder zur Entsperrung des Smartphones)						
**Chatbots** (z.B. ChatGPT, Freddy Freshbot von Hello Fresh, Amazon-Chat-Support)						

AI satisfaction, English translation

<u>Instruction:</u> Please indicate how satisfied you are with the use of the listed AI systems.

	satisfied	rather satisfied	rather dissatisfied	dissatisfied	never used	no response
**Virtual assistants** (e.g. Alexa, Google Assistant, Siri)						
**Route suggestions** (e.g. Google Maps)						
**Facial recognition** (e.g. on photos or for unlocking the smartphone)						
**Chatbots** (e.g. ChatGPT, Fredy Freshbot from Hello Fresh, Amazon-Chat-Support)						

Supplement File S2. Acceptance Scale

This supplement presents the scale assessing participants' acceptance of AI-supported tele-emergency medicine. Both the originally used German scale and the English translation are provided. The scale used is based on the Model for Ethical Evaluation of Socio-Technical Arrangements (MEESTAR)[1]. In the survey the order of the items was randomized for each participant to avoid sequence effects[2].

Acceptance Scale, German original

Instruktion: Teilen Sie uns bitte in der folgender Tabelle mit, inwieweit die angeführten Aussagen für Sie zutreffen.

		trifft voll und ganz zu	trifft eher zu	trifft eher nicht zu	trifft gar nicht zu	keine Angabe
FS	Ein KI-unterstützter Telenotarzt würde die Patienten schneller versorgen als einer ohne KI-Unterstützung.					
FS	Ein KI-unterstützter Telenotarzt würde die Patienten besser versorgen als einer ohne KI-Unterstützung.					
Sb	Ich würde lieber einen KI-unterstützten Telenotarzt wählen, anstatt einen ohne KI-Unterstützung.					
Sb	Ich würde mich von einem KI-unterstützten Telenotarzt behandeln lassen.					
S_B	Ich würde mich während einer Behandlung vom KI-unterstützten Telenotarzt sicherfühlen.					
S_B	Ein KI-unterstützter Telenotarzt würde weniger Behandlungsfehler machen als einer ohne KI-Unterstützung.					
S_K	Der Einsatz von KI wäre hilfreich für die Verordnung von Behandlungsmaßnahmen in der Notfallmedizin.					
S_K	Der Einsatz von KI wäre hilfreich für die Stellung von Diagnosen in der Notfallmedizin.					
S_D	Ein vertrauensvoller Umgang mit Daten wird durch den Einsatz von KI in der Notfallmedizin ermöglicht.					
Sv	Ich würde mir wünschen, dass Telenotärzte in der Zukunft mit Unterstützung durch eine KI arbeiten.					
Sv	Der Einsatz von KI in der Notfallmedizin würde zu einer besseren medizinischen Versorgung führen.					
G	Die Arbeit der Telenotärzte könnte durch den Einsatz von KI erleichtert werden.					

FS = Fürsorge; Sb = Selbstbestimmung; S_B = Sicherheit der Behandlung; S_K = Sicherheit, Kompetenz der KI; S_D = Sicherheit, Datensicherheit; Sv = Selbstverständnis; G = Gerechtigkeit;

[1] Manzeschke, A. MEESTAR: ein Model angewandter Ethik im Bereich assistiver Technologien [MEESTAR: a model of applied ethics in assistive technology]. In Technisierung des Alltags. Beitrag für ein gutes Leben?, Weber, K.; Frommeld, D.; Manzeschke, A.; Fangerau, H., Eds.; Franz Steiner Verlag: Stuttgart, Germany 2015; pp.263–283.
Manzeschke, A.; Weber, K.; Rother, E.; Fangerau, H. Ethische Fragen im Bereich Altersgerechter Assistenzsysteme: Er-gebnisse der Studie [Ethical Issues in the Field of Age-Appropriate Assistive Systems: Study Results]. VDI/VDE Innova-tion + Technik GmbH: Munich, Germany 2013, pp.3–38..

[2] Fröhl, U.; Friedrich, C. Quick Guide Onlinefragebogen - Wie Sie Ihre Zielgruppe professionell im Web befragen [Quick Guide Online Questionnaire - How to professionally survey your target group online]. Springer Gabler: Wiesbaden, Germany 2022; pp. 101-132.

Acceptance Scale, English translation

Instruction: Please indicate in the following table to what extent you agree with the listed statements.

		strongly agree	somewhat agree	somewhat disagree	strongly disagree	no response
C	An AI-supported tele-emergency physician would provide faster patient care compared to one without AI support.					
C	An AI-supported tele-emergency physician would provide better patient care compared to one without AI support.					
A	I would rather choose an AI-supported tele-emergency physician than one without AI support.					
A	I would let an AI-supported tele-emergency physician treat me.					
S_T	I would feel secure during treatment by an AI-supported tele-emergency physician.					
S_T	An AI-supported tele-emergency physician would make fewer treatment errors than one without AI support.					
S_C	The implementation of AI would be beneficial for prescribing treatment measures in emergency medicine.					
S_C	The implementation of AI would be beneficial for diagnosing patients in emergency medicine.					
S_D	The implementation of AI enables a trustworthy management of data in emergency medicine.					
SC	I would wish for tele-emergency physicians to be supported by AI in the future.					
SC	The implementation of AI in emergency medicine would lead to improved medical care.					
J	The implementation of AI would ease the work of tele-emergency physicians.					

C = Care; A = Autonomy; S_T = Therapy safety; S_C = Safety, AI competence; S_D = Safety, Data security; SC = Self-conception; J = Justice
